# Oral mucositis self-management ability among cancer survivors receiving radiotherapy or chemotherapy in China: A latent profile analysis

**DOI:** 10.1016/j.apjon.2025.100770

**Published:** 2025-08-06

**Authors:** Shanshan Zhang, Lin Wang, Juan Qiao, Hanfei Cui, Xuebing Jing

**Affiliations:** a Oncology Department, Zibo Central Hospital, Zibo, China; b Clinical Trial Center, Zibo Central Hospital, Zibo, China; c Hematology Department, Zibo Central Hospital, Zibo, China

**Keywords:** Cancer survivors, Oral mucositis, Self-management, Latent profile analysis

## Abstract

**Objective:**

This study aimed to investigate the self-management status of RIOM (Radiation-induced Oral Mucositis)/CIOM (Chemotherapy-induced Oral Mucositis) among cancer survivors in China, thereby uncovering latent profiles and predictors to guide supportive care interventions.

**Methods:**

Convenience sampling was conducted. A total of 399 cancer survivors who received radiotherapy/chemotherapy at a tertiary hospital in Zibo City between January and March 2025 were selected as the study participants. Using validated scales, we assessed the self-management ability of the RIOM/CIOM, social support (SSRS), and illness acceptance (AIS). Latent profile analysis (LPA) identified heterogeneous subgroups, and subsequent logistic regression analysis examined the associated factors.

**Results:**

Among the 399 enrolled cancer survivors, LPA stratified oral mucositis (OM) self-management ability into three distinct categories: Low Level-Negative self-management type (11.03%), Medium Level-Relatively self-management type (26.32%), High Level-Autonomous self-management type (62.66%). The key modifiable factors that were significantly associated (*P* ​< ​0.05) with OM self-management ability included psychosocial factors (disease acceptance and social support), behavioral factors (smoking status), and socioeconomic determinants (employment status, education level, marital status and monthly income).

**Conclusions:**

The self-management ability of cancer survivors with OM undergoing radiotherapy/chemotherapy was above average, with significant heterogeneity observed. Health care providers should implement personalized interventions tailored to the distinct self-management profiles of different patient subgroups to enhance their OM self-management capacity.

## Introduction

Malignant tumors are a global public health concern that severely threaten human health. According to the latest statistics from the World Health Organization's International Agency for Research on Cancer, cancer is the second leading cause of death worldwide.^1^ With the widespread adoption of early cancer screening technologies and the optimization of comprehensive treatment plans, the five-year survival rate of patients with cancer has significantly improved, transforming the disease attributes from fatal to chronic.^2^ Currently, radiotherapy and chemotherapy remain the cornerstone therapeutic approaches for cancer.^3^ However, although these treatments kill tumor cells, they often cause various treatment-related adverse effects, among which oral mucositis (OM) is particularly prominent.^4^ Evidence-based medical studies^5^^,^^6^ have indicated that the incidence of OM in patients with head and neck cancer receiving radiotherapy can reach 80% to 100%, whereas approximately 20% to 40% of patients undergoing conventional chemotherapy suffer from OM. OM is clinically characterized by oral mucosal erythema, erosion, ulcers accompanied by pain, and, in severe cases, dysphagia. These symptoms not only lead to inadequate nutritional intake and reduced quality of life (QoL), but may also cause treatment interruptions, ultimately affecting tumor control outcomes and increasing health care resource consumption.^4^

As the core theoretical framework of modern health management, the self-management theory emphasizes improving health outcomes by enhancing patient self-efficacy, self-regulation, and collaborative management.^7^ This theory holds significant value in chronic disease management. For instance, the Chronic Disease self-management Program proposed by Lorig and Holman^8^ highlights that patients need to master core skills such as symptom control, emotional management, and lifestyle adjustments. The Barlow's^9^ Self-Management Framework emphasizes the ability of patients to identify and solve health problems, effectively use medical and social resources, and make informed decisions based on health information. In the field of cancer rehabilitation, self-management is defined as a continuous process of collaboration between patients and health care providers to minimize treatment side effects and improve QoL. Multiple studies^10^, ^11^, ^12^ have established that well-developed self-management capabilities substantially reduce treatment-induced symptoms including cancer pain and nausea, while enhancing patients' QoL, self-efficacy, and overall physiological–psychological equilibrium. Particularly, for patients undergoing radiotherapy and chemotherapy, who typically experience short hospitalization cycles coupled with high demands for outpatient self-care, enhanced self-management ability can effectively compensate for diminished medical supervision during treatment intervals. Current evidence highlights the multifactorial nature of determinants influencing OM self-management. A systematic review^13^ revealed that although self-management interventions show efficacy in improving outcomes for patients with lung cancer, their benefits for OM remain primarily due to knowledge gaps encompassing disease pathophysiology, severity stratification, and evidence-based management protocols. Key implementation barriers were identified as follows: compromised physical functioning, psychological comorbidities (particularly anxiety and depression), and high symptom burden. A cross-sectional study involving 220 patients with cervical cancer^14^ revealed a significant association between overall self-management capacity (3.87 ​± ​0.53) and suboptimal symptom control (3.11 ​± ​0.82). A multivariate regression analysis identified three independent predictors: (1) socioeconomic status (quantified as per capita monthly household income), (2) treatment modality, and (3) social support networks. These findings imply that comprehensive family engagement and structured social support systems may substantially boost patients' motivation and adherence to self-management protocols. Another cross-sectional study involving 30 young cancer survivors (aged 18–29 years) undergoing chemotherapy^15^ found that despite possessing medication management knowledge, external support systems are still required. Therefore, strengthening OM self-monitoring and management abilities in patients undergoing radiotherapy/chemotherapy holds significant clinical value for early identification, timely intervention, and improved prognosis.

Current research on OM self-management ability in patients undergoing radiotherapy/chemotherapy primarily focuses on the status of self-management behaviors, effectiveness of interventions, role of self-efficacy, patient needs, family and social support, application of digital tools, long-term prognosis, and cross-cultural comparisons.^16^, ^17^, ^18^ Although Chinese scholars have recognized the importance of self-management in alleviating OM symptoms and improving the patients' QoL, its clinical application remains inadequate.^19^ First, most studies have concentrated solely on self-efficacy at the group level,^20^ lacking in-depth analysis of intra-group heterogeneity and failing to categorize patient subgroups according to efficacy levels. Second, although research has confirmed the multifaceted benefits of social support, such as alleviating psychological stress, enhancing self-efficacy and treatment adherence, and ultimately improving clinical outcomes, the underlying mechanisms remain insufficiently explored.^21^ Furthermore, disease acceptance, as a key psychological factor,^22^ has not been fully elucidated in terms of its correlation with pain tolerance and symptom management. Social support and disease acceptance are core elements influencing self-management abilities; robust social support can boost patients’ confidence and behavioral adherence and strong disease acceptance can enhance their ability to cope with treatment. However, research gaps remain in the following areas: the association between social support, disease acceptance, and latent categories of OM self-management abilities; personalized management needs of patient subgroups with different characteristics; and stratified intervention strategies based on LPA.

Latent profile analysis (LPA) is an advanced statistical method based on a person-centered approach. By establishing latent category models, participants are divided into several subgroups with distinct characteristics.^23^ Its core advantage lies in overcoming the limitations of the traditional group-mean analysis and providing methodological support for precision medicine. In OM self-management research, LPA has distinct advantages. This method can identify latent subgroups with different OM self-management characteristics, reveal heterogeneous patterns within groups, and provide a scientific basis for stratified interventions. It uncovers the patterns of heterogeneity within groups, thereby providing a solid scientific foundation for the implementation of stratified interventions. The findings aim to develop personalized management plans, optimize clinical screening standards, and facilitate a paradigm shift from“group intervention” to “precision intervention”, offering empirical evidence for clinical practice guidelines.

Therefore, this study aimed to categorize the self-management status of RIOM (Radiation-induced Oral Mucositis)/CIOM (Chemotherapy-induced Oral Mucositis) into different groups using LPA, and to explore the influencing factors that contribute to the differences between these groups. The findings of this study provide valuable insights for the development of targeted interventions for improving OM self-management among cancer survivors.

## Methods

### Study design

This study employed convenience sampling to recruit 418 cancer survivors who received radiotherapy/chemotherapy at a tertiary hospital in Zibo City from January to March 2025 as the study participants. Based on the sample size estimation method for analytical studies in cross-sectional research,^24^ the required sample size was at least 10 times the number of independent variables. The minimum sample size was 300 based on the 30 independent variables included in this study. Accounting for a 10% dropout rate, the required sample size was at least 330, and 418 patients were included in total.

### Inclusion and exclusion criteria

Inclusion criteria of the patients were as follows: (1) age ≥ 18 years; (2) pathologically confirmed malignant tumor; (3) inpatients who receiving radiotherapy, chemotherapy, or both; (4) expected survival ≥ 6 months; and (5) willingness to participate and signed informed consent. The exclusion criteria were as follows: (1) history of mental illness or cognitive impairment, (2) severe complications, and (3) inability to complete the questionnaire.

### Research tools

(1) General information questionnaire: age (years), sex, employment status, duration of illness (years), monthly income per capita (RMB), marital status, treatment modality, residence, education level, alcohol consumption, smoking history, surgical history, hypertension, diabetes, tumor stage, and mucositis grade.
(2) OM Self-Management Scale (SMS): Developed by Hanfei et al.^25^ in 2024, this tool assesses the self-management abilities of patients with cancer having OM. It includes four dimensions: medication management, oral pain management, disease monitoring and management, and daily life management, with a total of 15 items. A Likert five-point scale was used, with a maximum score of 75. Higher scores indicated stronger self-management abilities. The Cronbach's α coefficient of the scale was 0.902, and the test-retest reliability was 0.862.In this study, the Cronbach's α coefficient for this scale was 0.806.
(3) The Social Support Rating Scale (SSRS), Developed by Shuiyuanin 1986, this tool evaluates individuals' social support levels.^26^ It includes three dimensions: objective support, subjective support, and support utilization, with a total of 10 items. Items 1–4 and 8–10 use a Likert four-point scale, item 5 is scored from no support (one point) to full support (four points), and items 6 and 7 are scored based on the number of support sources (zero points for “no sources”). The total score ranges from 12 to 66, with higher scores indicating higher levels of social support. Scores of 45–66, 23–44, and ≤ 22 represent high, moderate, and low levels of support, respectively. The scale demonstrated excellent reliability, with a Cronbach's α coefficient of 0.920 and the test-retest reliability ranging from 0.890 to 0.940. In this study, the Cronbach's α coefficient for the SSRS was 0.816.
(4) Acceptance of Illness Scale (AIS): Developed by Felton^27^ in 1984 and translated into Chinese by Wenwenin 2018,^28^ the AIS assesses patients' acceptance of their illness. Its eight items cover four aspects: disease-related limitations, reduced self-care ability, dependence on others, and loss of confidence. A Likert five-point scale was used, with “strongly agree” scored as 1 and “strongly disagree” as 5. The total score ranged from 8 to 40, with 8–18, 19–29, and 30–40 indicating, low, moderate, and high acceptance, respectively. The Cronbach's α coefficient of the scale was 0.754. In this study, the Cronbach's α coefficient for the AIS was 0.834.

### Data collection

The research team included three oncology head nurses, two nursing team leaders, and two charge nurses. Before study initiation, team leaders trained members on participant selection and questionnaire collection techniques (e.g., standardized wording). All members completed the training and assessment. Two responsible nurses selected the subjects from four oncology wards according to the inclusion criteria and obtained informed consent from the subjects. They then distributed the QR code or paper questionnaire on Questionnaire Star. It took about 15–20 min to complete the questionnaire.After obtaining informed consent, the researchers distributed QR codes for the online questionnaires or the paper versions. Patients completed the questionnaires independently. For those with difficulties, the researchers recorded the answers verbatim. Questionnaires were collected on site and reviewed. To prevent duplicate filling, set the same IP address can only be submitted once.A total of 418 questionnaires were distributed with 399 valid responses (effective return rate of 95.45%). Missing values were addressed using multiple imputations.

### Statistical analysis

Mplus 8.3 software was used for LPA. Patients’ OM self-management abilities served as manifest variables. The initial model started with “C1”, and the number of latent categories was incrementally increased until optimal model fit was achieved. Model fit indices included the Akaike Information Criterion (AIC), Bayesian Information Criterion (BIC), and sample-adjusted BIC (aBIC), with smaller values indicating a better fit. Entropy assessed classification accuracy, with values closer to 1 indicating higher precision (≥ 0.8 suggests > 90% accuracy). The Lo-Mendell-Rubin adjusted likelihood ratio test (LMRT) and bootstrap likelihood ratio test (BLRT) were used to compare model fit differences, with *P* ​< ​0.05, indicating that the K-model fit was superior to that of the K-1 model. SPSS 26.0 was used for statistical analysis. Categorical data are presented as frequencies and percentages. Based on the optimal model, chi-square tests and one-way ANOVA compared differences among latent categories in general information, OM self-management abilities, disease acceptance, and social support. Multivariate logistic regression was used for multifactor analysis, with *P* ​< ​0.05 considered statistically significant.

## Results

### Demographic and clinical characteristics

A total of 418 questionnaires were distributed in this study. Following rigorous quality control procedures, 19 invalid responses were excluded based on predefined criteria: 1) Inconsistent responses (*n* ​= ​12): questionnaires containing logically contradictory answers; 2) Patterned responses (*n* ​= ​5): questionnaires exhibiting excessively uniform response patterns; 3) Short-duration responses (*n* ​= ​2): questionnaires completed in less than 3 minutes (substantially below the average completion time).

Among the 399 patients, ages ranged from 26 to 88 years (54.27 ​± ​14.93), and 209 (52.38%) and 190 (47.62%) were men and women, respectively. The details are presented in Table 1.

**Table 1:** Univariate analysis of the three latent profiles of OM self-management ability.

Variable	Group	*N* (%)	Low level	Medium level	High level	*χ**^2^**/F*	*P*
			(*n* ​= ​44)	*(n* ​= ​105)	(*n* ​= ​250)		
**Age (years)**	< 45	122 (30.58%)	24 (54.55%)	38 (36.19%)	60 (24.00%)	21.743	< 0.000∗
	45–60	146 (36.59%)	13 (29.55%)	40 (38.10%)	93 (37.20%)		
	≥ 60	131 (32.83%)	7 (15.91%)	27 (25.71%)	97 (38.80%)		
**Sex**	Male	209 (52.38%)	27 (61.36%)	65 (61.90%)	117 (46.80%)	8.363	0.015∗
	Female	190 (47.62%)	17 (38.64%)	40 (38.10%)	133 (53.20%)		
**Employment status**	Unemployed	65 (16.29%)	12 (27.27%)	16 (15.24%)	37 (14.80%)	24.547	< 0.000∗
	Employed	180 (45.11%)	22 (50.00%)	57 (54.29%)	101 (40.40%)		
	On leave	107 (26.82%)	9 (20.45%)	29 (27.62%)	69 (27.60%)		
	Retired	47 (11.78%)	1 (2.27%)	3 (2.86%)	43 (17.20%)		
**Duration of illness (years)**	< 3	127 (31.83%)	32 (72.73%)	41 (39.05%)	54 (21.60%)	93.080	< 0.000∗
	3–5	139 (34.84%)	11 (25.00%)	54 (51.43%)	74 (29.60%)		
	≥ 5	133 (33.33%)	1 (2.27%)	10 (9.52%)	122 (48.80%)		
**Monthly income (RMB)**	< 5000	59 (14.79%)	13 (29.55%)	14 (13.33%)	32 (12.80%)	24.504	< 0.000∗
	5000–10,000	118 (29.57%)	8 (18.18%)	47 (44.76%)	63 (25.20%)		
	> 10,000	222 (55.64%)	23 (52.27%)	44 (41.90%)	155 (62.00%)		
**Marital status**	Single	151 (37.84%)	23 (52.27%)	63 (60.00%)	65 (26.00%)	65.238	< 0.000∗
	Divorced/widowed	80 (20.05%)	15 (34.09%)	22 (20.95%)	43 (17.20%)		
	Married	168 (42.11%)	6 (13.64%)	20 (19.05%)	142 (56.80%)		
**Treatment modality**	Chemotherapy	95 (23.81%)	14 (31.82%)	40 (38.10%)	41 (16.40%)	21.660	< 0.000∗
	Radiotherapy	183 (45.86%)	19 (43.18%)	36 (34.29%)	128 (51.20%)		
	Both	121 (30.33%)	11 (25.00%)	29 (27.62%)	81 (32.40%)		
**Residence**	Rural	115 (28.82%)	22 (50.00%)	30 (28.57%)	63 (25.20%)	11.724	< 0.000∗
	Town	178 (44.61%)	13 (29.55%)	45 (42.86%)	120 (48.00%)		
	Urban	106 (26.57%)	9 (20.45%)	30 (28.57%)	67 (26.80%)		
**Education level**	High school	60 (15.04%)	14 (31.82%)	15 (14.29%)	31 (12.40%)	31.804	< 0.000∗
	College	108 (27.07%)	14 (31.82%)	39 (37.14%)	55 (22.00%)		
	Bachelor's	132 (33.08%)	14 (31.82%)	34 (32.38%)	84 (33.60%)		
	Graduate	99 (24.81%)	2 (4.55%)	17 (16.19%)	80 (32.00%)		
**Alcohol use**	No	244 (61.15%)	27 (61.36%)	62 (59.05%)	155 (62.00%)	0.272	0.873
	Yes	155 (38.85%)	17 (38.64%)	43 (40.95%)	95 (38.00%)		
**Smoking history**	No	247 (61.90%)	18 (40.91%)	66 (62.86%)	163 (65.20%)	9.416	0.009∗
	Yes	152 (38.10%)	26 (59.09%)	39 (37.14%)	87 (34.80%)		
**Surgical history**	No	316 (79.20%)	33 (75.00%)	84 (80.00%)	199 (79.60%)	0.536	0.765
	Yes	83 (20.80%)	11 (25.00%)	21 (20.00%)	51 (20.40%)		
**Hypertension**	No	303 (75.94%)	31 (70.45%)	87 (82.86%)	185 (74.00%)	3.989	0.136
	Yes	96 (24.06%)	13 (29.55%)	18 (17.14%)	65 (26.00%)		
**Diabetes**	No	289 (72.43%)	28 (63.64%)	79 (75.24%)	182 (72.80%)	2.136	0.344
	Yes	110 (27.57%)	16 (36.36%)	26 (24.76%)	68 (27.20%)		
**Tumor stage**	II	123 (30.83%)	12 (27.27%)	26 (24.76%)	85 (34.00%)	3.813	0.432
	III	130 (32.58%)	17 (38.64%)	37 (35.24%)	76 (30.40%)		
	IV	146 (36.59%)	15 (34.09%)	42 (40.00%)	89 (35.60%)		
**Mucositis grade**	Mild	128 (32.08%)	17 (38.64%)	30 (28.57%)	81 (32.40%)	2.262	0.688
	Moderate	117 (29.32%)	10 (22.73%)	35 (33.33%)	72 (28.80%)		
	Severe	154 (38.60%)	17 (38.64%)	40 (38.10%)	97 (38.80%)		
**Disease acceptance**			12.129 ​± ​3.569	19.695 ​± ​6.466	26.640 ​± ​5.911	139.959	< 0.000∗
**Social support**			18.454 ​± ​4.342	28.000 ​± ​7.704	36.904 ​± ​7.070	159.492	< 0.000∗

OM, oral mucositis. ∗*P* ​< ​0.05.

### LPA of OM self-management ability among cancer survivors

An exploratory analysis of OM self-management ability was conducted using the 1–5 latent profile models (Table 2). The fit indices showed that as the number of categories increased, the AIC and aBIC values decreased continuously. All models had entropy values ​> ​0.8, indicating good classification quality. The optimal model was determined as follows: The 3-class model had the highest entropy (0.865) and the LMR and BLRT *P* were ​< ​0.01. Although the 4-and 5-class models passed the statistical tests (*P* ​< ​0.05), their entropy values declined (0.809 and 0.802, respectively). Additionally, the 4-class model included a small probability category (< 10% of the sample). Therefore, considering model simplicity, clinical interpretability, and fit indices, the 3-class latent profile was selected as the optimal model.

**Table 2:** Fit indices for latent profile models of OM self-management ability (*N* ​= ​399).

Model	AIC	BIC	ABIC	LMRT	BLRT	Entropy	Classprobabilities
1	8407.960	8439.872	8414.487	–	–	–	–
2	8065.916	8117.772	8076.523	0.0003	0.0000	0.871	0.31/0.69
3	7982.975	8054.776	7997.661	0.0170	0.0000	0.865	0.11/0.63/0.26
4	7947.716	8039.462	7966.482	0.0234	0.0000	0.809	0.23/0.09/0.14/0.55
5	7923.487	8035.178	7946.333	0.2038	0.0000	0.802	0.08/0.16/0.13/0.08/0.55

OM, oral mucositis; AIC, Akaike Information Criterion; BIC, Bayesian Information Criterion; ABIC, adjusted BIC; LMRT, Lo-Mendell-Rubin adjusted likelihood ratio test; BLRT, bootstrap likelihood ratio test.

**Fig. 1:**
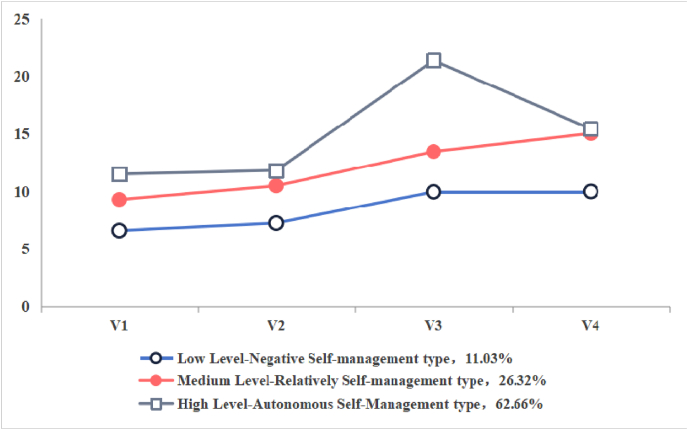
Distribution of characteristics in three potential categories of OM self-management ability among cancer survivors. OM, oral mucositis.

This study analyzes the characteristics of three potential categories of OM self-management ability by creating a line graph of scores for each dimension of OM self-management (Fig. 1). Based on the external features of each dimension of the scale, these categories are named as follows: Category 1 includes 44 cases (11.03%), where cancer survivors show low enthusiasm for OM self-management, particularly in medication and pain management, hence it is named the 'Low Level-Negative Self-management type'; Category 2 includes 105 cases (26.32%), where cancer survivors show relatively high enthusiasm for OM self-management, effectively monitoring disease progression and managing pain well, hence it is named the 'Medium Level-Relatively self-management type'; Category 3 includes 250 cases (62.66%), where cancer survivors can effectively manage OM, especially in comprehensive monitoring of their condition, hence it is named the 'High Level-Autonomous self-management type'.

### Univariate analysis of latent profiles in OM self-management ability

The results of the univariate analysis in Table 1 show that the three potential categories of the OM self-management ability of cancer survivors have statistically significant differences (*P* ​< ​0.05) in disease acceptance (*P* ​< ​0.000), social support (*P* ​< ​0.000), age (years) (*P* ​< ​0.000), sex (*P* ​= ​0.015), smoking (*P* ​= ​0.009), employment status (*P* ​< ​0.000), duration of illness (years) (*P* ​< ​0.000), marital status (*P* ​< ​0.000), residence (*P* ​< ​0.000), treatment modality (*P* ​< ​0.000), educational level (*P* ​< ​0.000), and monthly income (yuan) (*P* ​< ​0.000). There were no significant differences in alcohol consumption (*P* ​= ​0.873), surgical history (*P* ​= ​0.765), hypertension (*P* ​= ​0.136), diabetes (*P* ​= ​0.344), tumor stage (*P* ​= ​0.432), or OM grade (*P* ​= ​0.688) (*P* ​> ​0.05).

### Multivariate analysis of latent profiles in OM self-management ability

We used a multivariate logistic regression to analyze the factors influencing the self-management ability of cancer survivors in the OM domain. The three potential categories of OM self-management ability were used as dependent variables, and the statistically significant factors identified in the univariate analysis were included as independent variables in the regression analysis (for variable assignment, Table 3). The 'Low Level-Negative self-management type' was used as the control group to explore the factors affecting the OM self-management ability of each potential category, with specific results presented in Table 4.

**Table 3:** Variable assignment methods.

Independent Variable	Assignment method
Age	< 45 (Z_1_ ​= ​0, Z_2_ ​= ​0), 45–60 (Z_1_ ​= ​1, Z_2_ ​= ​0), ≥ 60 (Z_1_ ​= ​0, Z_2_ ​= ​1)
Employment status	Unemployed (Z_1_ ​= ​0, Z_2_ ​= ​0, Z_3_ ​= ​0), Employed (Z_1_ ​= ​1, Z_2_ ​= ​0, Z_3_ ​= ​0), On leave (Z_1_ ​= ​0, Z_2_ ​= ​1, Z_3_ ​= ​0), Retired (Z_1_ ​= ​0, Z_2_ ​= ​0, Z_3_ ​= ​1)
Duration of illness	< 3 years (Z_1_ ​= ​0, Z_2_ ​= ​0), 3–5 years (Z_1_ ​= ​1, Z_2_ ​= ​0), ≥ 5 years (Z_1_ ​= ​0, Z_2_ ​= ​1)
Monthly income	< 5000 (Z_1_ ​= ​0, Z_2_ ​= ​0), 5000–10,000 (Z_1_ ​= ​1, Z_2_ ​= ​0), > 10,000 (Z_1_ ​= ​0, Z_2_ ​= ​1)
Marital status	Single (Z_1_ ​= ​0, Z_2_ ​= ​0), Divorced/widowed (Z_1_ ​= ​1, Z_2_ ​= ​0), Married (Z_1_ ​= ​0, Z_2_ ​= ​1)
Residence	Rural (Z_1_ ​= ​0, Z_2_ ​= ​0), Town (Z_1_ ​= ​1, Z_2_ ​= ​0), Urban (Z_1_ ​= ​0, Z_2_ ​= ​1)
Education level	High school (Z_1_ ​= ​0, Z_2_ ​= ​0, Z_3_ ​= ​0), College (Z_1_ ​= ​1, Z_2_ ​= ​0, Z_3_ ​= ​0), Bachelor's (Z_1_ ​= ​0, Z_2_ ​= ​1, Z_3_ ​= ​0), Graduate (Z_1_ ​= ​0, Z_2_ ​= ​0, Z_3_ ​= ​1)
Treatment modality	Radiotherapy (Z_1_ ​= ​0, Z_2_ ​= ​0), Chemotherapy (Z_1_ ​= ​1, Z_2_ ​= ​0), Both (Z_1_ ​= ​0, Z_2_ ​= ​1)
Sex	Male ​= ​1, Female ​= ​0
Smoking	Yes ​= ​1, No ​= ​0
Disease acceptance	Raw score input
Social support	Raw score input

**Table 4:** Multiple Logistic regression analysis results of potential categories of OM self-management ability in cancer survivors.

Variable	Class 1 vs. Class 2					Class 1 vs. Class 3				
	β	OR	95% CI Low	95% CI High	*P*	β	OR	95% CI Low	95% CI High	*P*
**Disease acceptance**	0.389	1.475	1.016	2.141	0.041	0.412	1.511	1.015	2.248	0.042
**Social support**	0.164	1.178	0.929	1.495	0.176	0.321	1.378	1.057	1.796	0.018
**Sex**	−0.447	0.639	0.153	2.669	0.540	0.460	1.584	0.329	7.627	0.566
**Smoking history**	−2.016	0.133	0.028	0.637	0.012	−2.193	0.112	0.020	0.613	0.012
**Employment status**										
Employed	1.386	4.000	0.626	25.576	0.143	1.245	3.472	0.405	29.794	0.256
On leave	2.772	15.985	1.527	167.273	0.021	3.497	33.032	2.348	464.588	0.010
Retired	0.055	1.057	0.040	27.777	0.974	2.190	8.934	0.263	303.547	0.223
**Duration of illness (years)**										
3–5	1.988	7.301	1.582	33.703	0.011	2.215	9.158	1.657	50.608	0.011
≥ 5	2.563	12.978	0.572	294.624	0.108	5.302	200.671	8.045	5005.468	0.001
**Monthly income (RMB)**										
5000-10,000	2.186	8.900	1.106	71.640	0.040	1.447	4.248	0.406	44.480	0.227
> 10,000	1.195	3.303	0.597	18.259	0.171	1.749	5.748	0.790	41.828	0.084
**Marital status**										
Divorced/widowed	−2.086	0.124	0.024	0.644	0.013	−1.473	0.229	0.035	1.488	0.123
Married	−0.049	0.952	0.139	6.514	0.960	2.588	13.308	1.678	105.580	0.014
**Residence**										
Town	0.593	1.810	0.373	8.789	0.462	1.238	3.448	0.574	20.721	0.176
Urban	0.605	1.831	0.312	10.733	0.502	0.524	1.689	0.229	12.438	0.607
**Education level**										
College	−0.201	0.818	0.104	6.442	0.849	−1.096	0.334	0.032	3.537	0.362
Bachelor's	1.205	3.338	0.422	26.395	0.253	1.071	2.918	0.282	30.151	0.369
Graduate	1.865	6.457	0.417	100.012	0.182	3.049	21.089	1.064	418.135	0.045
**Age (years)**										
45–60	1.001	2.722	0.483	15.335	0.256	1.286	3.620	0.509	25.738	0.199
≥ 60	0.603	1.828	0.165	20.266	0.623	1.504	4.501	0.327	61.952	0.261
**Treatment modality**										
Chemotherapy	−0.576	0.562	0.115	2.747	0.477	0.765	2.148	0.357	12.929	0.404
Both	−0.416	0.660	0.109	3.977	0.650	0.299	1.348	0.180	10.073	0.771

OM, oral mucositis; OR, odds ratio; CI, confidence interval; ref, reference. Class 1 ​= ​Low Level-Negative self-management type; Class 2 ​= ​Medium Level-Relatively self-management type; Class 3 ​= ​High Level-Autonomous self-management type.

## Discussion

This study innovatively applied LPA combined with self-management theory to overcome the limitations of traditional variable-centered analysisin assessing OM self-management abilities.^29^ The results showed that the overall OM self-management abilities score among cancer survivors was 53.95 ​± ​10.435, indicating an above-average level. Using LPA, we identified three distinct OM self-management subgroups: Low Level-Negative self-management type (11.03%), Medium Level-Relatively self-management type (26.32%), High Level-Autonomous self-management type (62.66%). Multivariate logistic regression further revealed that the High Level-Autonomous self-management type was characterized by non-smoking status, being on leave, longer illness duration (≥ 5 years), being married, having graduate-level education, and higher disease acceptance and social support. Medium Level-Relatively self-management type was characterized by non-smoking status, being on leave, longer illness duration (3–5 years), Monthly income (5000–10,000 RMB) and higher disease acceptance. These findings provide critical evidence for the development of precise interventions for OM self-management.

Our research found that smoking cancer survivors are more likely to fall into the Low Level-Negative self-management type, consistent with the findings of Shiyu.^30^ Smoking not only increases the risk of oral cancer but also weakens OM self-management abilities through the following mechanisms: (1) nicotine and tar directly damage the oral mucosa, exacerbating inflammation and pain; and (2) smokers often have other unhealthy habits.^31^ Moreover, the smoking rate was significantly higher among those who were divorced, separated, or lost a spouse (38.7%) than among married individuals,^32^ and being Married was a protective factor for High Level-Autonomous self-management type, possibly due to health monitoring, emotional support, and joint decision-making by partners.^32^^,^^33^ This difference highlights the critical role of social support, with a previous study^34^ showing that social support is positively correlated with the ability to manage chronic diseases. Clinicians are recommended to adopt a step-by-step smoking cessation plan, create smoke-free environments, and visualize the harmful effects of smoking to improve compliance. For special populations such as those who have lost a spouse or are divorced, “peer support” groups, “family and friend supervision” systems, and psychological counseling services can be established to enhance social support and OM self-management skills.^34^

This study found that cancer survivors in the on leave were more likely to belong to the High Level-Autonomous self-management type. In-service patients tend to have weaker social support and self-management abilities because of reduced social participation and increased work stress.^35^^,^^36^ Kim^37^ confirmed that social support can alleviate anxiety and depression, enhancing psychological resilience. They recommend that clinicians seize the “vacation window”, reinforcing OM self-management skills through structured education, and establishing flexible work arrangements and online support systems.^36^^,^^37^ Additionally, controlling the potential impact of socioeconomic factors, such as income and educational level, is necessary.

The results of this study showed that the duration of illness was a significant factor influencing OM self-management abilities among cancer survivors. Patients with a disease course of 3–5 years were mostly in the middle and high-level groups, whereas those with a disease course of > 5 years were mainly in the high-level group. Long-term patients have the following advantages: (1) more effective use of social support resources, (2) establishment of a stable intrinsic motivation system,^38^ (3) accumulation of disease management experiences (understanding symptom patterns, optimizing care strategies, and refining medication decisions).^38^ In contrast, newly diagnosed patients often face limitations in OM self-management because of psychological distress (anxiety/depression/fear), insufficient understanding of their condition, and difficulties in emotional regulation.^39^

Our research shows that cancer survivors with graduate degrees are more likely to belong to the High Level-Autonomous self-management type, which is consistent with a previous study.^40^ One study^41^ found that, educational level is closely related to information seeking; patients with higher education levels have greater confidence in self-management. They leverage strong information retrieval and analysis skills to proactively integrate resources from multiple channels and form rational and systematic self-management models. Liu et al.^42^ found that the difference in educational level leads to a significant cognitive gap. Survivors with low education cannot identify the early symptoms of OM correctly and timely, and the rationality score of symptom coping strategies is also low, leading to the failure of timely intervention in the early stage of OM. Therefore, health care providers should pay more attention to patients with lower educational levels, develop illustrated tools for OM recognition, establish one-on-one health guidance, and use audiovisual media to simplify health education content, among other measures, to effectively address the differences in OM self-management capabilities caused by educational levels.^41^

This study found that patients in the “middle–high level” group exhibited significantly higher disease acceptance than those in the “low–level” group. Disease acceptance promotes self-management behaviors through three key pathways: improving treatment adherence, fostering positive health behaviors, and enhancing self-management efficacy.^43^^,^^44^ Notably, among cancer survivors who have undergone long-term chemotherapy and radiotherapy, disease acceptance has a unique value in alleviating body image distress and enhancing the QoL. Research based on the Swanson care theory framework^45^^,^^46^ confirmed that a four-stage intervention model of understanding, companionship, support, and empowerment can significantly improve patient disease acceptance. Therefore, it is recommended that clinical practice systematically assesses patients’ disease acceptance status, implements structured interventions based on care theory for those with low acceptance, and establishes a tripartite support network involving “patients, family members, and health care providers” to comprehensively enhance their OM self-management capabilities.

This study found that cancer survivors with higher social support scores were more likely to belong to the High Level-Autonomous self-management type. In the Medium Level-Relatively self-management type, social support was not statistically significant, which may be associated with the fact that the patients in this group mainly relied on passive compliance rather than active participation. Social support has both direct and indirect buffering effects on the health and emotional adjustment of cancer survivors, improving health behaviors by providing practical assistance, and alleviating symptoms by indirectly reducing psychological stress.^47^ A qualitative study^48^ found that, social support among cancer survivors is associated with decreased physical function, fatigue, pain, anxiety, and depression and can buffer the negative impact of stress practices on health through perceived support and family assistance. Specifically, low levels of social support increased the likelihood of low self-management among cancer survivors. Therefore, we recommend a systematic assessment of the disease acceptance status of patients in clinical practice. For patients with low acceptance, structured interventions based on care theory should be implemented, along with the establishment of a tripartite support network involving patients, family members, and health care providers to comprehensively enhance their OM self-management capabilities.

In the first univariate analysis, we found that age showed significant differences. However, in the final model, age was not an independent factor influencing OM self-management ability. The relationship between age and self-management ability has always been contradictory. Wenyu's study^49^ found that age and self-management ability have a significant negative correlation, whereas Akin et al. did not find this association.^50^ Although older patients experience some decline in cognitive function, this can be partially compensated for through accumulated experience, and management ability should not be predicted based solely on age. Given the limited evidence on the relationship between age and self-management ability in patients with OM, further research is required to verify these findings.

The results of this study showed that the treatment modality was statistically significant (*P* ​< ​0.000) with cancer survivors' OM self-management in univariate analysis, but after adjusting for confounding factors (Age/Employment status/Education level/Residence, etc.), this association was no longer statistically significant. This suggests that treatment may indirectly affect cancer survivors' OM self-management through age, education or Employment status, rather than as independent predictors. According to comprehensive evidence from domestic and international studies,^51^^,^^52^ the cancer survivors' OM self-management is more dependent on personal internal factors (such as health literacy and self-efficacy) and external support systems than directly determined by treatment methods. Treatment approaches may indirectly influence self-management behaviors by affecting symptom severity. The classification of treatment is too broad to mask key differences (such as mucosal toxicity differences between different chemotherapy drugs), which will need to be further validated with larger samples and more refined treatment classifications.

### Implications for nursing practice and research

This study focused on cancer survivors using LPA to examine the current status and factors influencing OM self-management abilities. The aim was to enhance health care providers “comprehensive understanding of cancer survivors” OM self-management abilities and to provide personalized support and interventions to encourage them to actively and collaboratively address the challenges of cancer. It is recommended that clinical medical staff pay particular attention to individuals with low disease acceptance, low social support, employment status, divorced or widowed marital status, smoking habits, and educational levels below junior high school as they exhibit lower self-management levels.

### Limitations

This study had some limitations.First, the cross-sectional design made it impossible to establish causality. Second, the study cohort was established with patients from only one hospital, which may have introduced a selection bias and affected the generalizability of the results. Additionally, there was insufficient control for potential confounding factors. This study did not consider the possible differences in the incidence of OM between patients with head and neck cancer and other cancers, and whether it affects patients' self-management ability is worth further discussion. However, the hospital selected for this study, a provincial-level regional medical center, has nearly 3500 beds actually in use. Of these, 400 are specialized oncology beds. Its medical service scope encompasses the city where it is located and four neighboring cities. It admits > 15,000 patients with cancer every year and is remarkably representative of the field of regional oncology diagnosis and treatment, which may reduce the bias caused by single-center sampling. Furthermore, there was insufficient control for potential confounding factors. Future research should adopt a longitudinal study design, increase the sample size, and further validate the findings of this study.

## Conclusions

This study categorized patients with OM related to radiotherapy and chemotherapy into three levels: Low Level-Negative self-management type, Medium Level-Relatively self-management type, High Level-Autonomous self-management type, through potential profile analysis, providing a basis for precise intervention. The results showed significant differences among the groups in dimensions such as disease acceptance and social support, with the Low Level-Negative self-management type requiring focused intervention. It is recommended that clinical practice implement stratified management, including maintaining existing support for the High Level-Autonomous self-management type, strengthening training for theHave we correctly interpreted the following funding source(s) and country names you cited in your article: Medium, United States? Medium Level-Relatively self-management type, and establishing a family collaboration system combined with cognitive behavioral therapy for the Low Level-Negative self-management type. Attention should also be paid to the moderating effects of high-impact factors, such as educational level and marital status. Future studies should focus on (1) validating the classification criteria of this study, (2) developing an AI automatic classification system, and (3) conducting randomized controlled trials of intervention programs tailored to different subgroups to improve patient QoL and treatment outcomes.

## CRediT authorship contribution statement

**SZ**: Conceptualization, Formal analysis, Methodology, Software, Validation, Visualization, Writing – Original draft. **LW**: Data curation, Investigation, Resources, Writing – Original draft. **JQ**: Data curation, Investigation, Resources, Writing – Original draft. **HC**: Data curation, Investigation, Resources, Writing – Original draft. **XJ**: Conceptualization, Project administration, Supervision, Writing – review & editing. All authors have read and approved the final manuscript.

## Ethics statement

The study was approved by the Medical Ethics Committee of Zibo Central Hospital (Approval No. 2025051) and was conducted in accordance with the Helsinki Declaration and its later amendments or comparable ethical standards. All participants provided written informed consent.

## Data availability statement

The data that support the findings of this study are available on request from the corresponding author, XJ, upon reasonable request.

## Declaration of generative AI and AI-assisted technologies in the writing process

No AI tools/services were used during the preparation of this work.

## Funding

This study received no external funding.

## Declaration of competing interest

The authors declared no conflict of interest.
